# Magnetocaloric Effect in Cu5-NIPA Molecular Magnet: A Theoretical Study

**DOI:** 10.3390/ma13020485

**Published:** 2020-01-19

**Authors:** Karol Szałowski, Pamela Kowalewska

**Affiliations:** Department of Solid State Physics, Faculty of Physics and Applied Informatics, University of Lodz, ulica Pomorska 149/153, PL90-236 Łódź, Poland

**Keywords:** molecular nanomagnets, magnetocaloric effect, magnetic entropy, magnetic specific heat, isothermal entropy change, magnetic cluster, Heisenberg model

## Abstract

We calculated the magnetocaloric properties of the molecular nanomagnet Cu5-NIPA, consisting of five spins S=1/2 arranged in two corner-sharing triangles (hourglass-like structure without magnetic frustration). The thermodynamics of the system in question was described using the quantum Heisenberg model solved within the field ensemble (canonical ensemble) using exact numerical diagonalization. The dependence of the magnetic entropy and magnetic specific heat on the temperature and the external magnetic field was investigated. The isothermal entropy change for a wide range of initial and final magnetic fields was discussed. Due to plateau-like behavior of the isothermal entropy change as a function of the temperature, a high degree of tunability of magnetocaloric effect with the initial and final magnetic field was demonstrated.

## 1. Introduction

Molecular magnets constitute a highly interesting class of modern magnetic materials [1,2], the rapid development of which over the last decades [3,4,5,6,7] required concerted effort of theoreticians and experimentalists. Zero-dimensional molecular nanomagnets [8,9] offer the possibility of exploring a plethora of intriguing fundamental physical phenomena due to the underlying quantum physics [10]. On the other hand, the possible multifunctionality of molecular magnets [11] opens the way towards numerous applications. Among them, the magnetic cooling based on the magnetocaloric effect (MCE) should be emphasized as particularly interesting application [12,13,14,15]. MCE [16] consists in the dependence of the entropy of substance on the external magnetic field and thus allows designing of a thermodynamic cycle for refrigeration (working between two constant temperatures) or outlining the procedure used to lower the temperature [17]. Therefore, MCE is a phenomenon of paramount importance for advanced and innovative cooling in diverse temperature ranges, from room temperature in everyday use to subkelvin range for experimental devices, which becomes a crucial technological challenge. The constant quest for optimized materials for exploiting MCE motivates studies of novel relevant materials [18]. This focuses the attention on molecular magnets as highly promising materials. One of their unique features is the direct applicability in nanoscale cooling. The molecular nanomagnets proved their potential in subkelvin cooling [19] and might be useful for on-chip cooling of nanoelectronic devices [20,21]. The quest for cooling efficiency stimulates the development of various approaches to design molecular magnets with desired properties [22,23,24,25,26]. Molecular magnets may offer record-high spin per molecule maximizing the potential span of entropy change, turning the attention to high spin clusters [27,28,29]. On the other hand, the maximization of MCE is searched in systems with magnetic frustration resulting from the interplay between the antiferromagnetic interactions and the geometry, where the relatively small changes of magnetic field can cause large variations of the magnetic entropy by lifting the quantum state degeneracy [30,31,32]. Another interesting route is utilizing the quantum level crossings [33]. In addition, such ideas as rotational MCE exploiting strong magnetic anisotropy [34,35] are investigated in molecular systems. These facts strongly motivate the interest in magnetocaloric properties of molecular magnets.

To understand and control MCE in molecular magnets, the development of theoretical models for description of their thermodynamics is of key importance [36]. For systems consisting of low enough number of spins, the exact methods for spin Hamiltonians can be applied. Therefore, in the context of the theoretical modeling of magnetic entropy and magnetocaloric properties of zero-dimensional systems, numerous works concerning spin clusters of various geometry can be mentioned first, especially those for spins 1/2. The exactly studied geometries include regular Ising polyhedra [37,38,39,40], planar Ising clusters based on the triangular lattice [41,42,43], or Ising clusters based on tetrahedra [44]. In addition to classical Ising model-based studies, quantum Heisenberg model has also been investigated by exact methods in the context of zero-dimensional cluster geometry, e.g., cube [45], a cuboctahedron [46], an edge-sharing tetrahedron [47], a hexagon [48], or a finite chain [49] clusters. Exact studies of Heisenberg model for particular molecular systems can also be found in the literature, e.g., a butterfly-shaped structure with higher spin [50] or our previous study concerning a structure with two interacting triangles [51]. The calculations become more even more demanding when structures involving high number of spins, especially S>1/2, are modeled. For this purpose, advanced and effective close-to-exact approaches for thermodynamic description of the spin systems are developed [52,53,54,55].

Among the basic cluster geometries, the triangle geometry as an underlying pattern for arrangement of spins can be highly interesting [56,57,58,59,60]. Its experimental realization in the form of quantum spin triangle can be based, for example, on Cu^2+^ ions which carry spin 1/2 and can form the relevant structures in molecular nanomagnets (see, e.g., [61,62,63,64,65,66]). The interplay of the triangular geometry and magnetic couplings between the spins may both lead to magnetic frustration or yield unfrustrated systems. Interacting triangles or more complex triangle-based structures can also be found in one-dimensional systems such as spin tubes or ladders, studied both theoretically [67,68] and experimentally [69,70].

However, a more complicated and rather uncommon form of triangle-based structure is a pair of corner-sharing triangles, possessing an hourglass-like geometry. Such a system is exemplified by a molecular magnet Cu_5_(OH)_2_(NIPA)_4_·10H_2_O (Cu5-NIPA) [71]. In this system, the couplings do not lead to magnetic frustration. Some of the magnetic properties of Cu5-NIPA were investigated as well as a theoretical model was constructed by Nath et al. [71]. Let us mention that somewhat similar, pentamer-based structures assembled of spin-1/2 copper ions are also reported [72,73,74,75], but they exhibit different magnetic interactions and geometry than Cu5-NIPA. The aim of our work was to characterize theoretically the magnetocaloric properties of the Cu5-NIPA nanomagnet. In the further sections, the theoretical model used to describe the MCE in Cu5-NIPA is outlined, the numerical results for the thermodynamic quantities of interest are discussed, and conclusions are drawn.

## 2. Theoretical Model and Computational Methods

**Figure 1:**
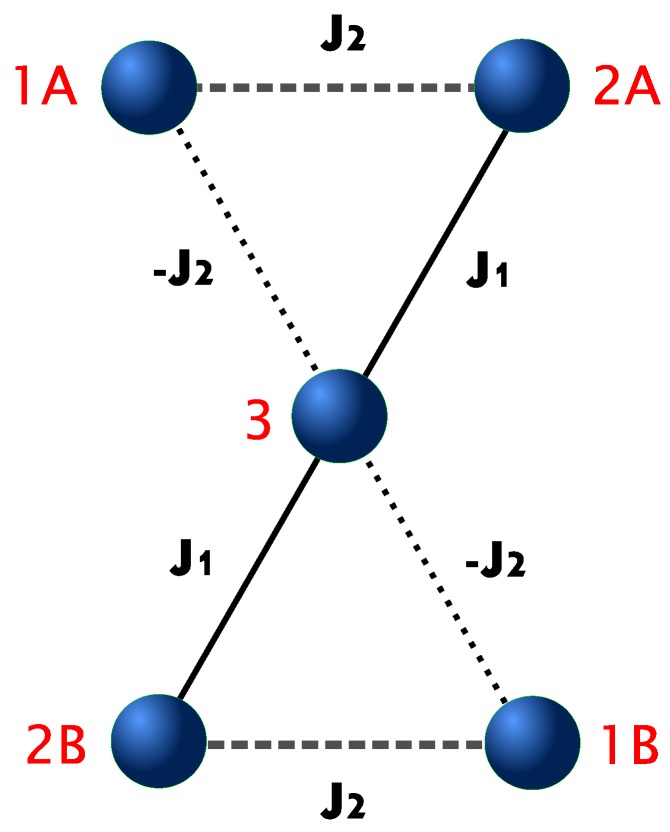
Schematic view of the Cu5-NIPA magnetic cluster with the relevant magnetic couplings marked.

The magnetic molecule of interest, Cu5-NIPA, contains five Cu ions carrying spins S=1/2 located in two corner-sharing triangles, forming an hourglass structure, as shown in Figure 1.

In our study we modeled the magnetic behavior of the system using an isotropic quantum Heisenberg model for spins

(1) $$\begin{array}{cc} \hat{H}= & -J_{1}\left(S_{2A}\cdot S_{3}+S_{2B}\cdot S_{3}\right)-J_{2}\left(S_{1A}\cdot S_{2A}+S_{1B}\cdot S_{2B}\right)+J_{2}\left(S_{1A}\cdot S_{3}+S_{1B}\cdot S_{3}\right)\\ & -g\mu_{B}B\left(S_{1A}^{z}+S_{1B}^{z}+S_{2A}^{z}+S_{2B}^{z}+S_{3}^{z}\right), \end{array}$$

where the operator $S_{i}=\left(S_{i}^{x},S_{i}^{y},S_{i}^{z}\right)$ is the operator of quantum spin S=1/2 situated in the hourglass-like structure at the site labeled with i= 1A, 1B, 2A, 2B, 3 (see Figure 1). In the system, there are four AF couplings (solid and dashed lines) and two F couplings (dotted lines), as indicated in Figure 1. The values of exchange integrals equal accordingly $J_{1}$ = −217 K and $J_{2}$ = −62 K (taken from the fitting to experimental data performed in Ref. [71]). Furthermore, *g* = 2.38 is the gyromagnetic factor, $\mu_{B}$ is Bohr magneton, and *B* is the external magnetic field, which is oriented along *z* direction. The Hamiltonian can be represented with a matrix of the size 32×32. Then, the total Hamiltonian undergoes the exact diagonalization (using Wolfram Mathematica software [76]), yielding complete set of eigenvalues and eigenvectors. The diagonalization was performed both analytically (see Appendix A) and numerically. The complete thermodynamic description was constructed using the canonical ensemble (actually in the version of field ensemble—please note that the term with external magnetic field is included in the Hamiltonian itself—see the discussion in Ref. [77]). The density operator is

(2) $$\hat{\rho }=\frac{1}{Z}\exp\left(-\frac{\hat{H}}{k_{B}T}\right),$$

where

(3) $$Z=\mathrm{Tr}\;\exp\left(-\frac{\hat{H}}{k_{B}T}\right)$$

is the statistical sum.

The knowledge of the statistical operator enables the calculation of the arbitrary thermodynamic averages. We mainly concentrate our attention on entropy, magnetic specific heat and isothermal entropy change. In particular, the Gibbs function (Gibbs free energy) is calculated as

(4) $$G=-k_{B}T\ln Z.$$

The quantity of fundamental interest is then the magnetic entropy *S*, expressed as

(5) $$S=\frac{U-G}{T},$$

where

(6) $$U=\mathrm{Tr}\;(\hat{\rho }\hat{H})$$

is the enthalpy. The magnetic specific heat is calculated from the relation

(7) $$c_{B}=\frac{\mathrm{Tr}\;(\hat{\rho }{\hat{H}}^{2})-{\left[\mathrm{Tr}\;(\hat{\rho }\hat{H})\right]}^{2}}{k_{B}T^{2}}.$$

The direct relation between the magnetic entropy and the magnetic specific heat is given by

(8) $$c_{B}=T{\left(\frac{\partial S}{\partial T}\right)}_{B}.$$

Let us mention that specific heat is a quantity directly measurable in the experiment as a function of the temperature and magnetic field. Therefore, it is used for determination of the entropy from the experimental data. Thus, the entropy at the given temperature and magnetic field can be expressed as the following integral:

(9) $$S\left(T,B\right)=\int_{0}^{T}\frac{c_{B}\left(T^{\prime },B\right)}{T^{\prime }}\;dT^{\prime }+S\left(T=0,B\right).$$

Let us emphasize that the integration constant is the ground-state entropy at T=0, which is given by $S\left(T=0,B\right)=k_{B}\ln g$, where *g* is the degeneracy of the ground state (see the discussion in Appendix A). On the other hand, the magnetic entropy in the limit of high temperature is determined by the number of states in the Hilbert space, that is $S\left(T\to \infty ,B\right)=k_{B}\ln{\left(2S+1\right)}^{n}$ for a system composed of *n* spins *S*. In the case of Cu5-NIPA composed of five spins S=1/2, it yields $S\left(T\to \infty ,B\right)=k_{B}\ln32=5\;k_{B}\ln2$. Therefore, the difference between entropies in the limit of high temperature and in the limit of zero temperature can be expressed on the basis of the following integral (see also [58]):

(10) $$S\left(T\to \infty ,B\right)-S\left(T=0,B\right)=k_{B}\ln\frac{{\left(2S+1\right)}^{n}}{g}=\int_{0}^{\infty }\frac{c_{B}\left(T,B\right)}{T}\;dT.$$

The isothermal entropy change, a fundamental quantity characterizing MCE, is expressed as

(11) $$\Delta S_{T}=S\left(T,B_{i}\right)-S\left(T,B_{f}\right),$$

being the change in entropy when the external field varies between $B_{i}$ and $B_{f}$ at constant temperature *T*. In this convention, $\Delta S_{T}>0$ corresponds to direct MCE, whereas $\Delta S_{T}<0$ implies the occurrence of inverse MCE.

The isothermal entropy change can be determined directly from the Equation (5) or, from the experimental data, using the integral in Equation (9) (see, e.g., [78]). Please note that, in the experiment, the ground-state entropy at T=0 is not evaluated directly.

Let us mention that the above thermodynamic formulas are valid for a single Cu5-NIPA cluster. Usually the corresponding thermodynamic quantities per mole are used and in the further presentation of the results of calculations such a convention is accepted.

In the subsequent part of the paper, we show and discuss our results, which we obtained using the exact numerical methods applied to the thermodynamic description of the model described with the Hamiltonian in Equation (1).

## 3. Results and Discussion

The present section contains the results of exact numerical calculations of the thermodynamic properties of Cu5-NIPA molecular magnet, performed along the lines described in Section 2. The interest is focused on the magnetocaloric properties, such as magnetic entropy, magnetic specific heat, and isothermal entropy change, which are expressed per mole of the substance of interest.

**Figure 2:**
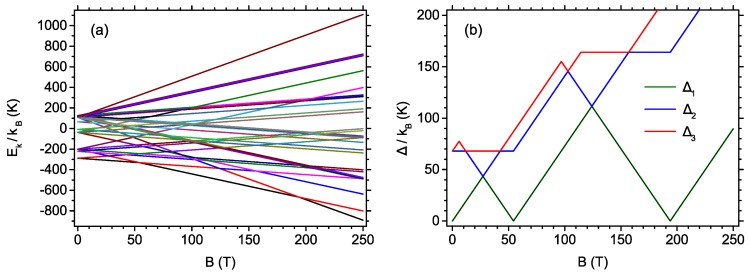
(**a**) The dependence of the energy levels for the system Hamiltonian on the external magnetic field; and (**b**) the dependence of the energy gaps between the ground state and a few first excited states on the external magnetic field.

Let us start the analysis by presenting the energy spectrum of the system Hamiltonian in Equation (1) as a function of the external magnetic field, which is shown in Figure 2a. A two-fold degeneracy of the ground state at B=0 is seen, whereas under the influence of B>0 the degeneracy is lifted. It can be deduced that three different quantum states can be (unique) ground states for B>0 and two critical fields (level-crossing fields) are present. The detailed analysis of the possible ground states and the analytic expressions for their energies is presented in Appendix A. In particular, below the first critical field of 54.36 T, the ground state has the total spin of S=1/2 and the spin projection quantum number is $S^{z}=1/2$. Between the first and the second critical field (which amounts to 193.91 T), the ground state has S=3/2 and $S^{z}=3/2$. Finally, above the second critical field, the magnetic saturation is reached, with S=5/2 and $S^{z}=5/2$. The analytic expressions for the critical fields as a function of the exchange integrals are also given in Appendix A. The fact that the energy levels cross when the external magnetic field is varied might be termed as quantum level crossing [79] (for example, in some analogy to the phenomenon emerging in a simplest system with interacting spins—a spin dimer [33,80], studied experimentally in the systems of various degree of complexity [80,81]). The change of the ground state of the system as a result of the variation of external parameter should induce the most profound consequences at zero temperature and at low temperatures, whereas the increase in *T* would smear this effect due to increased mixing of all states by thermal fluctuations.

The thermodynamic behavior of the system is to a significant extent governed by the energy gaps between the ground state and the first excited state (and further states). The dependence of the energy gaps $\Delta_{i}$ between the ground state and the *i*th excited state for i=1,2,3 is shown in Figure 2b. Between the field of 0 and 27.17 T, the most interesting gap $\Delta_{1}$ is a linearly increasing function of the field. It can be also seen that the second gap is usually significantly higher than the first gap (suggesting that the thermodynamics of the system in question can be Schottky model-like [38,51] and that the second excited state is usually well separated from the first excited state).

**Figure 3:**
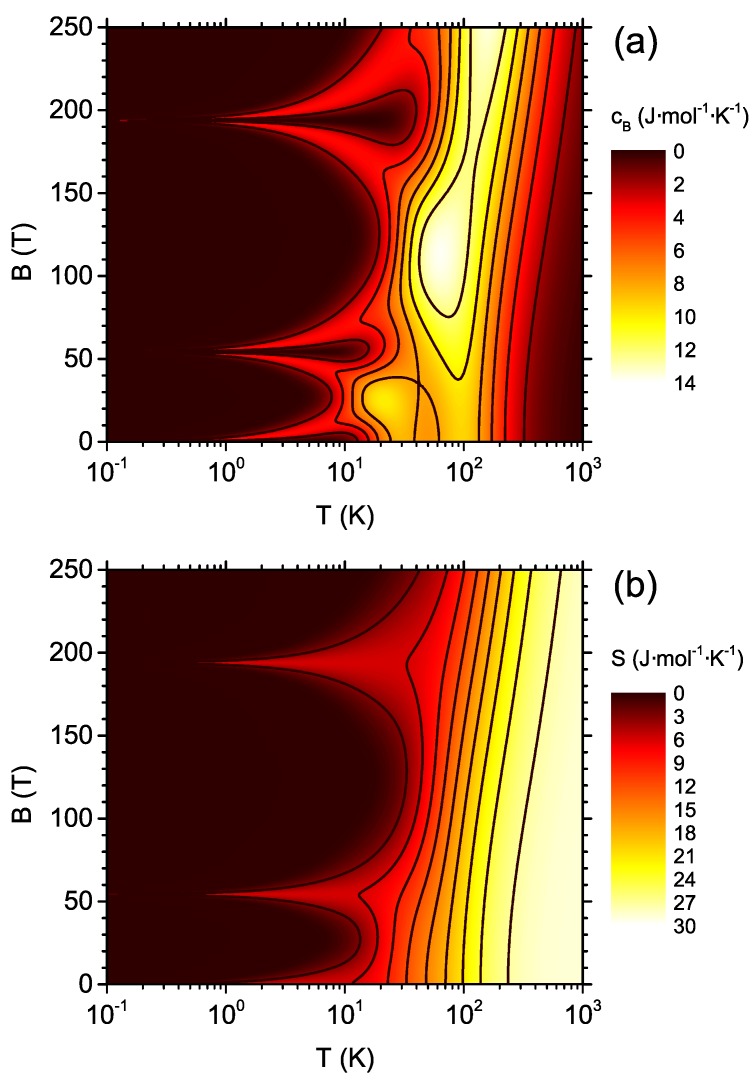
(**a**) The magnetic specific heat; and (**b**) the magnetic entropy as a function of the temperature and the external magnetic field. The features are visible in the vicinity of the quantum level crossings— for detailed discussion and critical field values, see Appendix A.

Let us commence the discussion of the thermodynamics of the system from the magnetic specific heat, which is a quantity measurable in the experiment. The density plot of the magnetic specific heat for a wide range of magnetic fields (in linear scale) and temperatures (in logarithmic scale) is presented in Figure 3a. In general, the greatest values of $c_{B}$ are noticed around 100 K, with a sort of maximum for considerably high magnetic fields. The position of the maximum is shifted towards higher temperatures when the field increases. For lower temperatures, three features are distinct. The first, single feature is present at low fields below the temperature of 10 K. Moreover, in the vicinity of both critical fields (level-crossing fields) where quantum level crossings occur (see Appendix A), clear double maxima separated by a deep minimum are visible and these maxima extend down to zero temperature. The overall temperature and field dependence of the magnetic specific heat is rather complicated.

For the analysis of the magnetocaloric properties, the crucial quantity of interest is the magnetic entropy as a function of the temperature and the magnetic field. The entropy density plot for a wide range of temperatures and magnetic fields is shown in Figure 3b (in logarithmic scale for the temperature). It is seen that the increase in the magnetic field causes the entropy to rise more slowly as a function of the temperature. The clear features in a form of single maxima are visible in the vicinity of the critical magnetic fields marking the quantum level crossings (see Appendix A), reflecting the positions of double maxima for the specific heat and also extending down to zero temperature.

**Figure 4:**
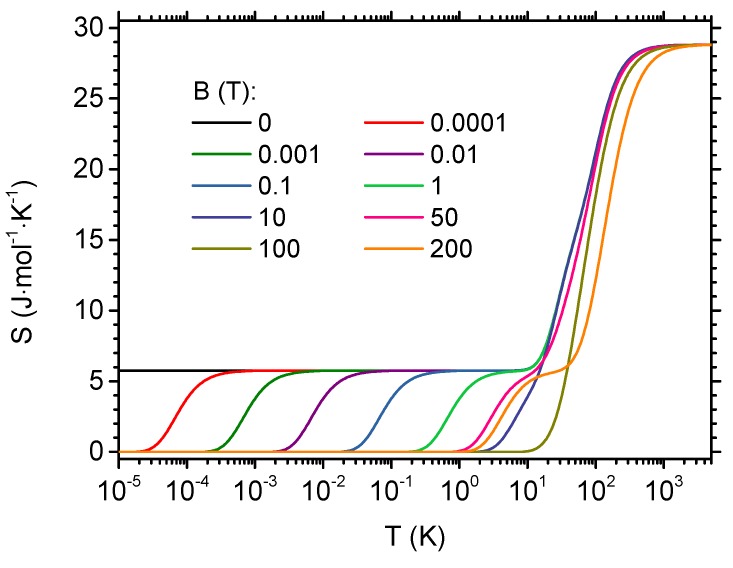
Entropy as a function of the temperature.

From the density plot in Figure 3b, a cross-section for constant magnetic fields showing the temperature dependence of the entropy can be constructed. Such a dependence is presented in Figure 4 in logarithmic scale for the temperature. For magnetic field equal to 0, the residual entropy of Rln2 is visible, due to two-fold degeneracy of the ground state at B=0 (the state with the total spin S=1/2 and projections onto *z* axis equal to ±1/2). The degeneracy is lifted for B>0. For the fields below approximately 10 T, the entropy rises first from 0 to Rln2 and then a plateau is present. At higher temperatures, the entropy rises to the saturation value of Rln32=5Rln2. The characteristic temperature at which the entropy jumps from 0 to Rln2 (the beginning of the plateau) increases when the magnetic field is increased. On the contrary, the temperature at which the plateau ends is rather insensitive to the field. It can be commented that the specific heat, $c_{B}=T{\left(\partial S/\partial T\right)}_{B}$, indicates a maximum due to this quite rapid change in entropy at the beginning of the plateau. This is the origin of the low-field and low-temperature feature seen in Figure 3a (for the detailed discussion, see the similar case in another molecular magnet investigated by us [51]). The behavior of the entropy for high fields, above 10 T, becomes much less regular.

**Figure 5:**
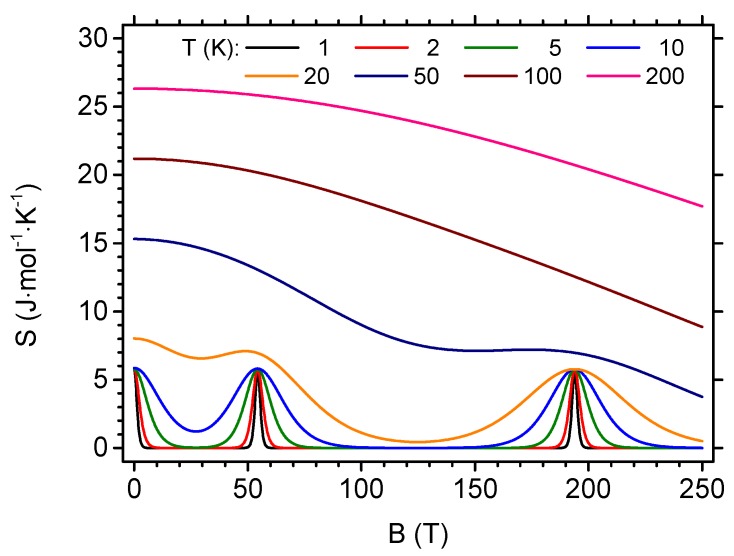
Entropy as a function of the magnetic field for various temperatures. The features are visible in the vicinity of the quantum level crossings—for detailed discussion and critical field values, see Appendix A.

Another interesting cross-section of Figure 3b is presented in Figure 5, where the entropy is plotted as a function of the magnetic field for different temperatures (entropy isotherms). When T→0, the entropy vanishes with the exceptions of the field B=0 (when the ground state with S=1/2 is two-fold degenerate) and both critical fields, $B_{c,1}$ and $B_{c,2}$ (where two energy states with different total spin quantum number *S* cross). At the mentioned points, the entropy value is, therefore, equal to the residual ground state entropy of Rln2. If the temperature rises, significant maxima build up around these values of the magnetic field and become smeared by the influence of increasing temperature. Up to considerably high temperature, the entropy is not a monotonic function of the field.

**Figure 6:**
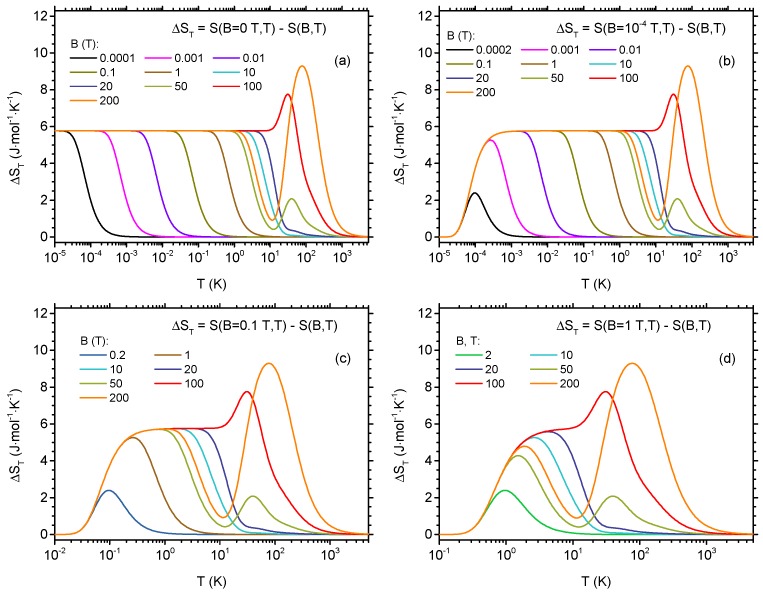
Isothermal entropy change as a function of temperature between the initial magnetic field and varying values of final magnetic field: (**a**) initial field of 0 T; (**b**) initial field of $10^{-}{}^{4}$ T; (**c**) initial field of 0.1 T; and (**d**) initial field of 1 T.

To characterize MCE in the studied Cu5-NIPA molecular magnet, we concentrate on the isothermal entropy change between the initial and final field given by Equation (11). Due to the residual entropy at T=0 it might be useful to study the entropy changes between various finite fields, not limiting the interest to the usual case of either initial or final field equal to zero. The first case, with initial field equal to zero and a selection of final fields, is shown in Figure 6a (please note the logarithmic temperature scale). All the curves start from Rln2 due to degeneracy of the ground state at B=0. The plateau of this height is present in the results for the final field not exceeding 10 T. When the final field increases, a plateau ends at increasingly higher temperatures, whereas its height remains unchanged. For very high final fields, a second maximum builds up at higher temperatures. The evolution of $\Delta S_{T}$ for non-zero initial field of 10${}^{-4}$ T and a selection of final fields can be followed in Figure 6b (note that the degeneracy of the ground state is now lifted already at initial field). In this case, for lowest final fields, a maximum builds up first and then the plateau develops. The overall dependence of $\Delta S_{T}$ on the temperature has a step-like shape with relatively sharp increase at some initial temperature and fall at some final temperature, with constant height. A similar behavior can be seen for the larger initial field of 0.1 T, as shown in Figure 6c, with a plateau commencing at higher temperature than for the previous case. If the initial field is as large as 1 T (Figure 6d), the temperature dependence of $\Delta S_{T}$ becomes rounded and resembles a moving peak.

**Figure 7:**
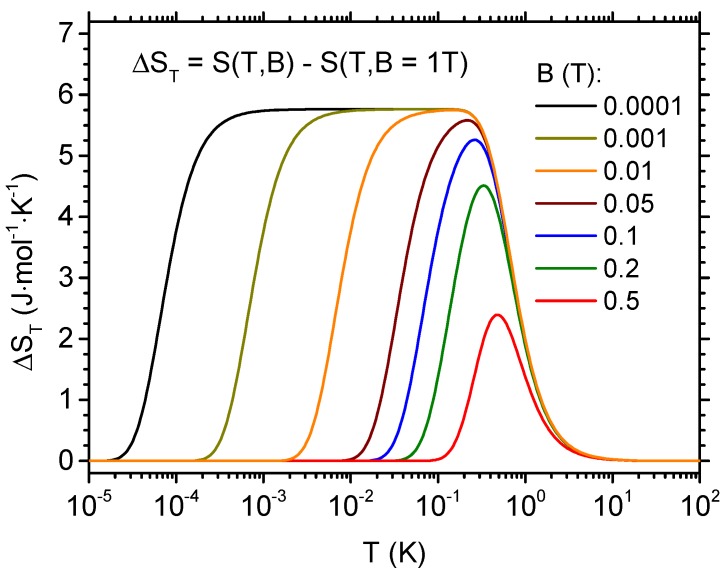
Isothermal entropy change as a function of temperature between the varying values of initial magnetic field and the final magnetic field of 1 T.

The tunability of the step-like temperature dependence of $\Delta S_{T}$ can be traced in detail in Figure 7, where the final field of 1 T is fixed and the cases of various values of the initial magnetic field are compared. In the range of initial fields down to 0.1 T, a peak builds up with only a slight tendency of shifting towards lower temperatures. If the initial field is below 0.1 T, a plateau in the temperature dependence of $\Delta S_{T}$ is developed and essentially the right end of this plateau remains at constant position. The left end shifts towards lower temperatures when the initial field is reduced.

**Figure 8:**
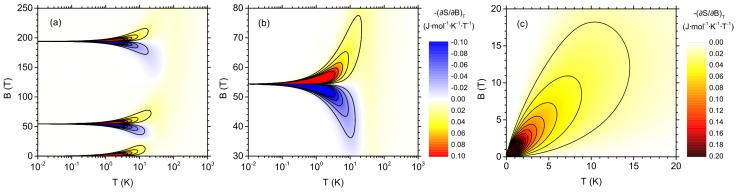
Differential entropy change, $-{\left(\partial S/\partial B\right)}_{T}$, as a function of the temperature and the magnetic field. Lines of constant differential entropy change are plotted: (**a**) full range of temperature and magnetic field (in logarithmic temperature scale and linear field scale); (**b**) magnetic fields in the vicinity of the lower critical field (in logarithmic temperature scale and linear field scale); and (**c**) range of weaker magnetic fields (in linear temperature and field scale). The features are visible in the vicinity of the quantum level crossings—for detailed discussion and critical field values, see Appendix A.

The magnetocaloric phenomena can be quantified not only by considering the isothermal entropy change under finite variation of the magnetic field $\Delta S_{T}$ discussed above. A supplementary quantity is a differential entropy change, characterized by ${\left(\partial S/\partial B\right)}_{T}$. The negative value of the derivative corresponds to direct MCE (i.e., the entropy decreases if the magnetic field rises), whereas inverse MCE means the positive value of the derivative. As a consequence, $-{\left(\partial S/\partial B\right)}_{T}$ is the convenient quantity to consider. The behavior of $-{\left(\partial S/\partial B\right)}_{T}$ is illustrated in Figure 8 as a function of the temperature and the magnetic field. The full range of *T* and *B* is depicted in Figure 8a (note the logarithmic scale for the temperature). In general, the most pronounced absolute values of the derivative are observed in the vicinity of the critical magnetic fields (where quantum level crossings occur) or close to the zero field. This is consistent with the behavior seen in Figure 5. For the fields just below $B_{c,1}$ or $B_{c,2}$ inverse MCE can be detected, while just above both critical fields the effect switches to direct MCE. The magnified range of parameters close to $B_{c,1}$ is shown in Figure 8b. The traces of the mentioned behavior can be noticed up to significant temperature, up to a few tens of K. The low-temperature feature in $-{\left(\partial S/\partial B\right)}_{T}$ is shown in detail in Figure 8c (this time in fully linear scale). The characteristic field at which the entropy derivative takes the maximum value is proportional to the temperature. This is related to the Schottky-like thermodynamic behavior of the system at low fields (when just two states—the ground state and the first excited state—are important). This is the regime where the energy gap between the states is proportional to the field and the characteristic temperature is proportional to the gap. These factors explain the described linear relation.

## 4. Final Remarks

In this paper, we present the calculation of the magnetocaloric properties of Cu5-NIPA molecular magnet—an unfrustrated system based on a pair of corner-sharing spin triangles. The modeling was based on the quantum Heisenberg Hamiltonian with exchange integrals taken from the experiment (see [71]). The field ensemble (version of the canonical ensemble) and exact numerical diagonalization was used for construction of the thermodynamic description and for calculation of the magnetic entropy as a function of the temperature and external magnetic field.

It was found that the behavior of such quantities as the magnetic entropy and specific heat as a function of the magnetic field is profoundly influenced by the crossing of energy levels corresponding to different total spins, since two such critical magnetic fields are present in the system. The pronounced maxima of entropy emerge in their vicinity and the derivative of entropy with respect to the magnetic field also shows peaks there. The thermodynamics of Cu5-NIPA for weaker magnetic fields is ruled by the Schottky-like behavior, with first excited state well separated from the higher excited states and the energy gap being a linear function of the field.

A persistent entropy plateau at the value of Rln2≃5.76 J·mol^−1^·K^−1^ is seen in the temperature dependence of the entropy at low temperatures. This causes the isothermal entropy change to take a step-like temperature dependence also with a similar plateau at the same value. The position of the beginning and the end of this plateau can be tuned with the initial or final magnetic field. Such a tunability concerns a rather wide span of temperatures (in general, belonging to subkelvin range, up to approximately a few K for the used magnetic fields not exceeding a few T). Therefore, the isothermal entropy change exhibits a rather high degree on tunability with the initial and final magnetic field used in the isothermal process. This feature might be used to optimize the behavior for the required temperatures for MCE applications. It is worth emphasizing that the maximum value of the entropy change per mole when exploiting the mentioned plateau is $\Delta S_{T}=R\ln2≃5.76$ J·mol^−1^·K^−1^. The value itself is not a record one, due to considerably low number of spins in the molecule and their low magnitude. However, a highly interesting feature is the flat plateau in the temperature dependence and step-like shape of the curve. This might contribute to rather high refrigerant capacity (being an integral of $\Delta S_{T}$ over the temperatures between the temperature of cool and hot reservoir in the thermodynamic cycle—see, for example, [82]). It can be noticed that the typical shape of the $\Delta S_{T}$ dependence on the temperature exhibits a single triangular peak and the working range of temperatures covers usually the full width at half maximum of the peak. The plateau-like shape is predicted for Cu5-NIPA for the range of initial and final fields not exceeding approximately 1 T. Somehow larger magnitudes of isothermal entropy change are obtainable for much higher fields, the achievement of which constitutes a highly challenging task. In particular, exploiting the full possible entropy change of 5Rln2≃28.81 J·mol^−1^·K^−1^ would require using the fields exceeding the second critical field $B_{c,2}$ of approximately 194 T.

The overall behavior of the system bears some resemblance to another molecular magnet, vanadium-based V6 (with two weakly interacting unfrustrated spin triangles), for which we studied similar aspects of MCE in Ref. [51]. This proves that the triangle-based systems with quantum spins S=1/2 might constitute highly tuneable MCE molecular materials. The further studies would include, for example, the calculation of some inequilibrium MCE properties. We also hope that our work may serve as a motivation for experimental study of thermodynamic properties of the Cu5-NIPA molecular system aimed at characterization of MCE (for example, in a similar way as in the case of vanadium-based molecules [78]). Finally, it might be mentioned that Cu5-NIPA as a magnetocaloric material does not contain rare-earth elements.

## Abbreviations

The following abbreviations are used in this manuscript:

MCE	Magnetocaloric effect
NIPA	5-nitro-isophthalic acid ligand
Cu5-NIPA	Cu_5_(OH)_2_(NIPA)_4_·10H_2_O

## Appendix A. The Ground States and Critical Magnetic Fields

For the molecular magnet Cu5-NIPA considered in the present work, the three states with different total spin *S* and total spin projection $S^{z}$ can constitute ground states in various ranges of the external magnetic field (for the values of exchange integrals given in Section 2). When the magnetic field is varied, quantum level crossings occur between the mentioned states. The energies of these states as well as the critical fields (level-crossing fields) corresponding to the crossings between them are discussed below. These energies were determined from exact diagonalization of the Hamiltonian (Equation (1)). The corresponding matrix size was 32×32 and the symbolic diagonalization was performed using Wolfram Mathematica software [76].

The energy of the state with S=1/2 and $S^{z}=1/2$ can be calculated as

(A1) $$E_{1/2}=\tilde{E}-\frac{1}{2}g_{e}\mu_{B}B,$$

where $\tilde{E}$ is one of the roots of the following cubic equation:

(A2) $${\tilde{E}}^{3}+\left(\frac{1}{2}J_{1}+J_{2}\right){\tilde{E}}^{2}-\left(\frac{1}{2}J_{1}^{2}+\frac{1}{2}J_{1}J_{2}+\frac{5}{4}J_{2}^{2}\right)\tilde{E}-\frac{3}{8}J_{1}J_{2}=0.$$

This root is expressed in closed form by the formula:

(A3) $$\tilde{E}=\frac{1}{2}\left(-1+i\sqrt{3}\right)\sqrt[{3}]{\frac{p}{2}+\sqrt{\frac{p^{2}}{4}+\frac{q^{3}}{27}}}-\frac{1}{2}\left(1+i\sqrt{3}\right)\sqrt[{3}]{\frac{p}{2}-\sqrt{\frac{p^{2}}{4}+\frac{q^{3}}{27}}},$$

where

(A4) $$p=\frac{1}{12}\left(7J_{1}^{2}+10J_{1}J_{2}+19J_{2}^{2}\right)$$

and

(A5) $$q=\frac{1}{108}\left(10J_{1}^{3}+33J_{1}^{2}J_{2}+12J_{1}J_{2}^{2}+53J_{2}^{3}\right).$$

Approximately, the energy can be expressed in the form of the power series in ratio $J_{2}/J_{1}$:

(A6) $$\tilde{E}≃J_{1}\left[1+\frac{J_{2}}{J_{1}}+\frac{7}{12}{\left(\frac{J_{2}}{J_{1}}\right)}^{2}-\frac{5}{36}{\left(\frac{J_{2}}{J_{1}}\right)}^{3}-\frac{103}{432}{\left(\frac{J_{2}}{J_{1}}\right)}^{4}+\frac{247}{1296}{\left(\frac{J_{2}}{J_{1}}\right)}^{5}+\frac{409}{2592}{\left(\frac{J_{2}}{J_{1}}\right)}^{6}+\ldots \right].$$

The energy of the state with S=3/2 and $S^{z}=3/2$ equals

(A7) $$E_{3/2}=\frac{1}{4}J_{1}-\frac{1}{4}J_{2}-\frac{\sqrt{3}}{4}J_{1}\sqrt{3-2\frac{J_{2}}{J_{1}}+7{\left(\frac{J_{2}}{J_{1}}\right)}^{2}}-\frac{3}{2}g_{e}\mu_{B}B.$$

The energy of the state with S=5/2 and $S^{z}=5/2$ equals

(A8) $$E_{5/2}=-\frac{1}{2}J_{1}-\frac{5}{2}g_{e}\mu_{B}B.$$

In the range of fields between 0 and $B_{c,1}$, the ground state has S=1/2, $S^{z}=1/2$ and the energy $E_{1/2}$. The critical field $B_{c,1}$ corresponds to the change to ground state with S=3/2, $S^{z}=3/2$ and can be calculated from the cubic equation resulting from the condition $E_{1/2}=E_{3/2}$. Approximately, it can be expressed as:

(A9) $$B_{c,1}≃\frac{|J_{1}|}{g_{e}\mu_{B}}\left[\frac{3}{2}\frac{J_{2}}{J_{1}}-\frac{1}{4}{\left(\frac{J_{2}}{J_{1}}\right)}^{2}-\frac{5}{12}{\left(\frac{J_{2}}{J_{1}}\right)}^{3}+\frac{19}{144}{\left(\frac{J_{2}}{J_{1}}\right)}^{4}+\frac{269}{432}{\left(\frac{J_{2}}{J_{1}}\right)}^{5}-\frac{151}{2592}{\left(\frac{J_{2}}{J_{1}}\right)}^{6}+\ldots \right].$$

The value of this critical field amounts to 54.36 T for the exchange integral values used in the present paper.

The second critical field, $B_{c,2}$, corresponds to the change of the ground state from S=3/2, $S^{z}=3/2$ to S=5/2, $S^{z}=5/2$ (i.e., the saturation state). Its value can be expressed by:

(A10) $$B_{c,2}=\frac{1}{4}\frac{|J_{1}|}{g_{e}\mu_{B}}\left[3-\frac{J_{2}}{J_{1}}+\sqrt{9-6\frac{J_{2}}{J_{1}}+21{\left(\frac{J_{2}}{J_{1}}\right)}^{2}}\right],$$

giving the value of 193.91 T for the Hamiltonian parameters used.

For the most interesting range of lower magnetic fields, the energy gap between the ground state and the first excited state, $\Delta_{1}$, is between the states with $S^{z}=1/2$ and $S^{z}=-1/2$, both with S=1/2. Therefore, it equals to:

(A11) $$\Delta_{1}=g_{e}\mu_{B}B.$$

At the field of 27.17 T, the first excited state becomes a state with S=3/2, $S^{z}=3/2$.

## References

[1] Pinkowicz D., Chorąży S., Stefańczyk O. (2011). An Invitation to Molecular Magnetism. Sci. Prog..

[2] Sieklucka B., Pinkowicz D. (2017). Molecular Magnetic Materials: Concepts and Applications.

[3] Blundell S.J., Pratt F.L. (2004). Organic and Molecular Magnets. J. Phys. Condens. Matter.

[4] Gatteschi D., Sessoli R. (2004). Molecular Nanomagnets: The First 10 Years. J. Magn. Magn. Mater..

[5] Blundell S.J. (2007). Molecular Magnets. Contemp. Phys..

[6] Schnack J. (2019). Large Magnetic Molecules and What We Learn from Them. Contemp. Phys..

[7] Coronado E. (2019). Molecular Magnetism: From Chemical Design to Spin Control in Molecules, Materials and Devices. Nat. Rev. Mater..

[8] Friedman J.R., Sarachik M.P. (2010). Single-Molecule Nanomagnets. Ann. Rev. Condens. Matter Phys..

[9] Hołyńska M. (2018). Introduction to Single-Molecule Magnets. Single-Molecule Magnets.

[10] Bertaina S., Gambarelli S., Mitra T., Tsukerblat B., Müller A., Barbara B. (2008). Quantum Oscillations in a Molecular Magnet. Nature.

[11] Fitta M., Pełka R., Konieczny P., Bałanda M. (2019). Multifunctional Molecular Magnets: Magnetocaloric Effect in Octacyanometallates. Crystals.

[12] Evangelisti M., Luis F., de Jongh L.J., Affronte M. (2006). Magnetothermal Properties of Molecule-Based Materials. J. Mater. Chem..

[13] Sessoli R. (2012). Chilling with Magnetic Molecules. Angew. Chem. Int. Ed..

[14] Zheng Y.Z., Zhou G.J., Zheng Z., Winpenny R.E.P. (2014). Molecule-Based Magnetic Coolers. Chem. Soc. Rev..

[15] Evangelisti M., Bartolomé J., Luis F., Fernández J.F. (2014). Molecule-Based Magnetic Coolers: Measurement, Design and Application. Molecular Magnets: Physics and Applications.

[16] Tishin A.M., Spichkin Y.I. (2003). The Magnetocaloric Effect and its Applications.

[17] Romero Gómez J., Ferreiro Garcia R., De Miguel Catoira A., Romero Gómez M. (2013). Magnetocaloric effect: A review of the thermodynamic cycles in magnetic refrigeration. Renew. Sustain. Energy Rev..

[18] Franco V., Blázquez J.S., Ipus J.J., Law J.Y., Moreno-Ramírez L.M., Conde A. (2018). Magnetocaloric effect: From materials research to refrigeration devices. Prog. Mater. Sci..

[19] Sharples J.W., Collison D., McInnes E.J.L., Schnack J., Palacios E., Evangelisti M. (2014). Quantum Signatures of a Molecular Nanomagnet in Direct Magnetocaloric Measurements. Nat. Commun..

[20] Ciccarelli C., Campion R.P., Gallagher B.L., Ferguson A.J. (2016). Intrinsic Magnetic Refrigeration of a Single Electron Transistor. Appl. Phys. Lett..

[21] Bradley D.I., Guénault A.M., Gunnarsson D., Haley R.P., Holt S., Jones A.T., Pashkin Y.A., Penttilä J., Prance J.R., Prunnila M. (2017). On-Chip Magnetic Cooling of a Nanoelectronic Device. Sci. Rep..

[22] Affronte M., Ghirri A., Carretta S., Amoretti G., Piligkos S., Timco G.A., Winpenny R.E.P. (2004). Engineering Molecular Rings for Magnetocaloric Effect. Appl. Phys. Lett..

[23] Evangelisti M., Brechin E.K. (2010). Recipes for Enhanced Molecular Cooling. Dalton Trans..

[24] Garlatti E., Carretta S., Schnack J., Amoretti G., Santini P. (2013). Theoretical Design of Molecular Nanomagnets for Magnetic Refrigeration. Appl. Phys. Lett..

[25] Liu J.L., Chen Y.C., Guo F.S., Tong M.L. (2014). Recent Advances in the Design of Magnetic Molecules for Use as Cryogenic Magnetic Coolants. Coord. Chem. Rev..

[26] Holleis L., Shivaram B.S., Balachandran P.V. (2019). Machine Learning Guided Design of Single-Molecule Magnets for Magnetocaloric Applications. Appl. Phys. Lett..

[27] Torres F., Hernández J.M., Bohigas X., Tejada J. (2000). Giant and Time-Dependent Magnetocaloric Effect in High-Spin Molecular Magnets. Appl. Phys. Lett..

[28] Gajewski M., Pełka R., Fitta M., Miyazaki Y., Nakazawa Y., Bałanda M., Reczyński M., Nowicka B., Sieklucka B. (2016). Magnetocaloric Effect of High-Spin Cluster with Ni_9_W_6_ Core. J. Magn. Magn. Mater..

[29] Chen W.-P., Qin L., Camón A., Engelhardt L., Luis F., Winpenny R.E.P., Zheng Y.-Z. (2018). Quantum Monte Carlo simulations of a giant {Ni_21_Gd_20_} cage with a *S*=91 spin ground state. Nat. Commun..

[30] Schnack J. (2006). Frustration Effects in Magnetic Molecules. J. Low Temp. Phys..

[31] Schnack J., Schmidt R., Richter J. (2007). Enhanced Magnetocaloric Effect in Frustrated Magnetic Molecules with Icosahedral Symmetry. Phys. Rev. B.

[32] Pakhira S., Mazumdar C., Ranganathan R., Avdeev M. (2017). Magnetic Frustration Induced Large Magnetocaloric Effect in the Absence of Long Range Magnetic Order. Sci. Rep..

[33] Chakraborty T., Mitra C. (2019). Magnetocaloric Effect as a Signature of Quantum Level-Crossing for a Spin-Gapped System. J. Phys. Condens. Matter.

[34] Lorusso G., Roubeau O., Evangelisti M. (2016). Rotating Magnetocaloric Effect in an Anisotropic Molecular Dimer. Angew. Chem. Int. Ed..

[35] Beckmann C., Ehrens J., Schnack J. (2019). Rotational Magnetocaloric Effect of Anisotropic Giant-Spin Molecular Magnets. J. Magn. Magn. Mater..

[36] Engelhardt L., Luban M. (2010). Simple Models and Powerful Tools for Seeking a Comprehensive Understanding of the Magnetic Properties of Molecular Magnets. Dalton Trans..

[37] Strečka J., Karľová K., Madaras T. (2015). Giant Magnetocaloric Effect, Magnetization Plateaux and Jumps of the Regular Ising Polyhedra. Phys. B Condens. Matter.

[38] Karľová K., Strečka J., Madaras T. (2016). The Schottky-Type Specific Heat as an Indicator of Relative Degeneracy between Ground and First-Excited States: The Case Study of Regular Ising Polyhedra. Phys. B Condens. Matter.

[39] Karľová K., Strečka J., Richter J. (2017). Enhanced Magnetocaloric Effect in the Proximity of Magnetization Steps and Jumps of Spin-1/2 XXZ Heisenberg Regular Polyhedra. J. Phys. Condens. Matter.

[40] Karľová K., Strečka J., Madaras T. (2017). Isothermal Entropy Change and Adiabatic Change of Temperature of the Antiferromagnetic Spin-1/2 Ising Octahedron and Dodecahedron. Acta Phys. Pol. A.

[41] Žukovič M., Bobák A. (2014). Entropy of Spin Clusters with Frustrated Geometry. Phys. Lett. A.

[42] Žukovič M. (2015). Thermodynamic and Magnetocaloric Properties of Geometrically Frustrated Ising Nanoclusters. J. Magn. Magn. Mater..

[43] Žukovič M., Semjan M. (2018). Magnetization Process and Magnetocaloric Effect in Geometrically Frustrated Ising Antiferromagnet and Spin Ice Models on a ‘Star of David’ Nanocluster. J. Magn. Magn. Mater..

[44] Mohylna M., Žukovič M. (2019). Magnetocaloric Properties of Frustrated Tetrahedra-Based Spin Nanoclusters. Phys. Lett. A.

[45] Strečka J., Čisárová J. (2014). Magnetization Process and Adiabatic Demagnetization of the Antiferromagnetic Spin-1/2 Heisenberg Cubic Cluster. Acta Phys. Pol. A.

[46] Karľová K., Strečka J. (2017). Magnetization process and magnetocaloric effect of the spin-1/2 XXZ Heisenberg cuboctahedron. J. Low. Temp. Phys..

[47] Strečka J., Karľová K. (2018). Magnetization Curves and Low-Temperature Thermodynamics of Two Spin-1/2 Heisenberg Edge-Shared Tetrahedra. AIP Adv..

[48] Deb M., Ghosh A.K. (2016). Studies of Magnetocaloric Effect on Spin-1/2 J_1_-J_2_ Heisenberg Hexagons. AIP Conf. Proc..

[49] Haldar S., Ramasesha S. (2020). Magnetocaloric effect in molecular spin clusters and their assemblies: Exact and Monte Carlo studies using exact cluster eigenstates. J. Magn. Magn. Mater..

[50] Arian Zad H., Kenna R., Ananikian N. (2020). Magnetic and Thermodynamic Properties of the Octanuclear Nickel Phosphonate-Based Cage. Phys. A Stat. Mech. Appl..

[51] Kowalewska P., Szałowski K. (2020). Magnetocaloric Properties of V6 Molecular Magnet. J. Magn. Magn. Mater..

[52] Schnack J., Hage P., Schmidt H.J. (2008). Efficient Implementation of the Lanczos Method for Magnetic Systems. J. Comput. Phys..

[53] Schnack J., Ummethum J. (2013). Advanced Quantum Methods for the Largest Magnetic Molecules. Polyhedron.

[54] Ummethum J., Schnack J., Läuchli A.M. (2013). Large-Scale Numerical Investigations of the Antiferromagnetic Heisenberg Icosidodecahedron. J. Magn. Magn. Mater..

[55] Hanebaum O., Schnack J. (2015). Thermodynamic Observables of Mn_12_-Acetate Calculated for the Full Spin Hamiltonian. Phys. Rev. B.

[56] Mentrup D., Schmidt H.J., Schnack J., Luban M. (2000). Transition from Quantum to Classical Heisenberg Trimers: Thermodynamics and Time Correlation Functions. Phys. A Stat. Mech. Appl..

[57] Luban M., Borsa F., Bud’ko S., Canfield P., Jun S., Jung J.K., Kögerler P., Mentrup D., Müller A., Modler R. (2002). Heisenberg Spin Triangles in {V_6_}-Type Magnetic Molecules: Experiment and Theory. Phys. Rev. B.

[58] Haraldsen J.T., Barnes T., Musfeldt J.L. (2005). Neutron Scattering and Magnetic Observables for *S*=1/2 Spin Clusters and Molecular Magnets. Phys. Rev. B.

[59] Schmidt H.J. (2013). The General Spin Triangle. Int. J. Modern Phys. B.

[60] Brumfield A., Haraldsen J.T. (2019). Thermodynamics and Magnetic Excitations in Quantum Spin Trimers: Applications for the Understanding of Molecular Magnets. Crystals.

[61] Angaridis P.A., Baran P., Boča R., Cervantes-Lee F., Haase W., Mezei G., Raptis R.G., Werner R. (2002). Synthesis and Structural Characterization of Trinuclear Cu^II^-Pyrazolato Complexes Containing *μ*_3_-OH, *μ*_3_-O, and *μ*_3_-Cl Ligands. Magnetic Susceptibility Study of [PPN]_2_[(*μ*_3_-O)Cu_3_(*μ*-pz)_3_Cl_3_]. Inorg. Chem..

[62] Iida K., Qiu Y., Sato T.J. (2011). Dzyaloshinsky-Moriya Interaction and Long Lifetime of the Spin State in the Cu_3_ Triangular Spin Cluster by Inelastic Neutron Scattering Measurements. Phys. Rev. B.

[63] Ponomaryov A.N., Kim N., Jang Z.H., van Tol J., Koo H.J., Law J.M., Suh B.J., Yoon S., Choi K.Y. (2015). Spin Decoherence Processes in the S = 1/2 Scalene Triangular Cluster (Cu3(OH)). New J. Phys..

[64] Spielberg E.T., Gilb A., Plaul D., Geibig D., Hornig D., Schuch D., Buchholz A., Ardavan A., Plass W. (2015). A Spin-Frustrated Trinuclear Copper Complex Based on Triaminoguanidine with an Energetically Well-Separated Degenerate Ground State. Inorg. Chem..

[65] Kintzel B., Böhme M., Liu J., Burkhardt A., Mrozek J., Buchholz A., Ardavan A., Plass W. (2018). Molecular Electronic Spin Qubits from a Spin-Frustrated Trinuclear Copper Complex. Chem. Commun..

[66] Liu J., Mrozek J., Myers W.K., Timco G.A., Winpenny R.E.P., Kintzel B., Plass W., Ardavan A. (2019). Electric Field Control of Spins in Molecular Magnets. Phys. Rev. Lett..

[67] Alécio R.C., Lyra M.L., Strečka J. (2016). Ground States, Magnetization Plateaus and Bipartite Entanglement of Frustrated Spin-1/2 Ising-Heisenberg and Heisenberg Triangular Tubes. J. Magn. Magn. Mater..

[68] Zad H.A., Ananikian N., Kenna R. (2019). The Specific Heat and Magnetic Properties of Two Species of Spin-1/2 Ladders with Butterfly-Shaped Unit Blocks. J. Phys. Condens. Matter.

[69] Schnack J., Nojiri H., Kögerler P., Cooper G.J.T., Cronin L. (2004). Magnetic Characterization of the Frustrated Three-Leg Ladder Compound [(CuCl_2_ tach H)_3_Cl]Cl_2_. Phys. Rev. B.

[70] Ivanov N.B., Schnack J., Schnalle R., Richter J., Kögerler P., Newton G.N., Cronin L., Oshima Y., Nojiri H. (2010). Heat Capacity Reveals the Physics of a Frustrated Spin Tube. Phys. Rev. Lett..

[71] Nath R., Tsirlin A.A., Khuntia P., Janson O., Förster T., Padmanabhan M., Li J., Skourski Y., Baenitz M., Rosner H. (2013). Magnetization and Spin Dynamics of the Spin *S*=1/2 Hourglass Nanomagnet Cu_5_(OH)_2_(NIPA)_4_·10H_2_O. Phys. Rev. B.

[72] Bi L.-H., Kortz U. (2004). Synthesis and Structure of the Pentacopper(II) Substituted Tungstosilicate [Cu_5_(OH)_4_(H_2_O)_2_(A-*α*-SiW_9_O_33_)_2_]^10−^. Inorg. Chem..

[73] Nellutla S., van Tol J., Dalal N.S., Bi L.H., Kortz U., Keita B., Nadjo L., Khitrov G.A., Marshall A.G. (2005). Magnetism, Electron Paramagnetic Resonance, Electrochemistry, and Mass Spectrometry of the Pentacopper(II)- Substituted Tungstosilicate [Cu_5_(OH)_4_(H_2_O)_2_(A-*α*-SiW_9_O_33_)_2_]^10−^, A Model Five-Spin Frustrated Cluster. Inorg. Chem..

[74] Ishikawa R., Nakano M., Fuyuhiro A., Takeuchi T., Kimura S., Kashiwagi T., Hagiwara M., Kindo K., Kaizaki S., Kawata S. (2010). Construction of a Novel Topological Frustrated System: A Frustrated Metal Cluster in a Helical Space. Chem. Eur. J..

[75] Liu Z.-Y., Chu J., Ding B., Zhao X.-J., Yang E.-C. (2011). A novel $Cu_{5}^{II}$ cluster-based 3D magnetic framework with an overall *S*=1/2 spin ground state. Inorg. Chem. Commun..

[76] (2010). Mathematica, Version 8.0.4.

[77] Plascak J.A. (2018). Ensemble Thermodynamic Potentials of Magnetic Systems. J. Magn. Magn. Mater..

[78] Fu Z., Xiao Y., Su Y., Zheng Y., Kögerler P., Brückel T. (2015). Low-lying magnetic excitations and magnetocaloric effect of molecular magnet K_6_[V_15_As_6_O_42_(H_2_O)]·8H_2_O. EPL.

[79] Waldmann O. (2007). Field-induced level crossings in spin clusters: Thermodynamics and magnetoelastic instability. Phys. Rev. B.

[80] Chakraborty T., Singh H., Mitra C. (2015). Experimental evidences of singlet to triplet transition in a spin cluster compound. J. Magn. Magn. Mater..

[81] Julien M.-H., Jang Z.H., Lascialfari A., Borsa F., Horvatič M., Caneschi A., Gatteschi D. (1999). Proton NMR for Measuring Quantum Level Crossing in the Magnetic Molecular Ring Fe10. Phys. Rev. Lett..

[82] Gschneidner K.A., Pecharsky V.K. (2000). Magnetocaloric Materials. Ann. Rev. Mater. Sci..

